# Veterinary Jurisprudence

**Published:** 1890-09

**Authors:** 


### VETERINARY JURISPRUDENCE.

### THE DEHORNING OF CATTLE—TEST PROSECUTION AT KELLS.[1]

WE insert, by special request, the subjoined report of a case of prosecution for dehorning cattle in Ireland. The report is somewhat condensed from the *Farmers' Gazette* and the *Irish Times*, but the professional evidence is given in full, as published in the *Times.*

Mr. F. B. Henn, R.M., and Mr. John Keating, J.P., sat July 4th, and two following days, in the courthouse, Kells, for the purpose of adjudicating upon a test case concerning the dehorning of cattle, brought by the Crown under the act for the prevention of cruelty to animals.

The summons, which was at the instance of District Inspector Newland against James McDonagh, charged the defendant with having caused the horns of certain oxen—to wit: twenty-six oxen—to be cut off, and with having otherwise cruelly ill-treated the said oxen on the 17th day of April, 1890, at Carlanstown, in the county of Meath.

*Counsel for the Prosecution*—Mr. George Hart (instructed by Mr. George Keogh, Crown Solicitor).

*For the Defendant*—Mr. John Ross and Mr. J. Kenny, M.P. (instructed by Messrs. Reeves & Son, Dublin).

The defendant admitted the acts complained of, but pleaded justification.

### PROFESSIONAL EVIDENCE FOR THE PROSECUTION.

Professor Walley, Principal of the Royal (Dick) College of Edinburgh, and member of the Royal College of Veterinary Surgeons, deposed that he had made the subject of the dehorning of cattle a study for fourteen years. He produced a drawing of the section of the horn, made by himself. In young animals there was no horn. It was a secondary growth beginning after two months. When it began its growth it was not attached to the skull, and by cutting it you only cut the skin at the base of the horn. You could move the horn about with the fingers then, but afterward it got firmer. He then gave an explanation of the structure of the horn, and stated that the cutting of it off caused intense pain. The result was there was always some fever, and frequently severe fever. From his knowledge of the nerves and bloodvessels of the horn he knew that the operation caused great agony. The removal of the horn would tend to conceal coarseness of breeding, and it was done for that purpose in some cases. Proper surgical treatment immediately after the operation would minimise the pain. If only the tip of the horn was cut off, as was done in Scotland, no pain would be caused, as the bone would not be touched, and the animals would not gore one another. In his opinion there was no justification for this cruelty. If breeders only knew their interests in this country, they

[1] The Veterinarian, August, 1890.

would get polled bulls; 85 per cent. of their progeny were without horns. He mentioned that the practice of putting knobs on horns would serve the purpose of dehorning. If, however, dehorning must be performed, it should be when the animal was very young. The fact that an animal ate or chewed the cud after the operation was no evidence that pain and suffering were not caused.

*Cross-examined by Mr. Ross:* The operation was cruel and unnecessary. The after-effects caused greater pain than the operation. The polled Angus cattle were not so good for dairy purposes.

Professor William Pritchard, ex-President of the Royal College of Veterinary Surgeons, London, deposed that he considered dehorning cruel and unnecessary. If the horns were tipped with brass knobs, butting and goring could be prevented. He would advocate a more general breeding and rearing of polled cattle. He knew that dehorning was not practiced in the midland counties of England. The meat of the animal was not improved by dehorning, nor was the animal improved in any respect. The object of dehorning was to prevent the animals injuring each other. The operation produced agony and inflammation of that part of the head, which lasted several days.

*Cross-examined by Mr. Kenny:* Cattle had, to his knowledge, died from having been dehorned.

Professor John Woodroffe Hill, of Downton Agricultural College, deposed that he had studied the subject of dehorning, and, in his opinion, it was both a brutal and unnecessary operation. In England it was not practiced. The operation as described in this case was decidedly cruel. He would say that such an operation was more painful than the tearing out of a human nail. As the horn was secreted from a very sensitive structure, even a sharp blow on the horn was very painful. In some instances where cattle were dehorned and the cavities filled with tow and tar, matter or pus would exude if pressed with the finger. One object in dehorning in such cases was to pass off the animal as a polled one. The practice was also carried out to make a greater number of cattle fit in a smaller space.

*Cross-examined by Mr. Ross:* He thought it unnecessary to dock a horse, as he condemned all sorts of mutilation. Dehorning would not add to the quantity of flesh on the animal, nor would it improve the quality. He did not consider that dehorning was justified by thirty shillings or a pound more being given for polled animals. His opinion on the subject would not be altered by hearing that to abandon dehorning would cost Ireland £400,000 a year.

Mr. Francis Frederick Collins, veterinary surgeon for thirty-eight years, thirty of which were passed in the army, deposed that he considered dehorning caused great agony. It was believed by some persons that bullocks grew larger after having been dehorned. In some instances it was practiced to conceal the coarse breed or age of an animal.

*Cross-examined by Mr. Kenny:* He believed that "tipping" was as effective as dehorning.

*Re-examined:* He did not think that dehorning intrinsically improved the animals. If two animals of the same age were reared together—the one dehorned and the other with his horns—there would be no difference between the animals, provided they were of the same breed and their feeding was the same.

Professor McQueen, of the Royal Veterinary College, Camdentown, and Professor J. W. Axe gave similar testimony. This closed the case for the prosecution.

SECOND DAY.

Mr. Ross, having addressed the bench for the defense, called Professor Wallace, Professor of Agriculture, Edinburgh University. He deposed, in reply to Mr. Kenny, that he had frequently removed the horns of animals, and had seen them removed. He believed that the pain produced was only momentary. The operation should not be performed by a veterinary surgeon necessarily, but by a skilled man. He had seen it done in from three to ten seconds. The budding horns of calves should be removed by a sharp knife, cutting into skin and hair to prevent the growth of "scurs" or imperfect horns. When adult cattle were being dehorned, he disapproved of the use of the knife. The saw only should be used, and thereby a second operation was prevented. The advantages to be derived from dehorning were that animals did not abuse each other; that they settled quietly to feed, and further, the meat of animals dehorned was superior, although the scientific reason has not yet been ascertained. They had only commenced three years ago to dehorn cattle at Chicago, and the percentage was now increasing. Ten years ago there was not a dehorned animal there. The reason, he believed, was because polled cattle could be shipped along with cattle of other breeds which had been dehorned. Up to that time an enormous number of cattle had been lost by being gored, and he thought the amount of money now saved compensated for the pain suffered by the animals. In the Madras Presidency young cattle were dehorned by a red-hot iron being applied to the tip of the budding horn; only during the operation acute pain lasted. They ate well afterward and continued to thrive without a check. He did not approve of tipping the horns, although ripped wounds might thereby be prevented. Still the flesh of the animal butted would be bruised. Knobs were commonly put on cows, but they were never put on bullocks except for ornamental purposes. It was painful for the animal to butt with a knob or ball on its horn, and it also might cause the animal to slough or cast the horn. At the cattle stations in Australia very many cattle were injured, as he had seen, and he thought it would be well to dehorn cattle there. When the horns were removed the animals became more peaceful and throve better. He did not consider that the crossing of the pure-bred polled cattle with ordinary horned cattle would produce a breed within the present generation that would give satisfaction. The dehorned cattle were worth about thirty shillings more than horned cattle, because they fed better. Dehorning was increasing in England and Scotland, spreading from those places where the people had had experience of polled cattle. Bellowing was as often the result of fear as of pain. The court adjourned.

THIRD DAY.

Mr. Peter A. Lawlor, V.S., Dublin, deposed that during a practice of fourteen years he had seen thousands of dehorned cattle. He had seen the operation performed. He did not consider it very painful. The pain was substantially over when the operation was performed. He did not consider any treatment necessary after the operation. Horned cattle injured one another, but dehorned ones did not, and they fed better. When milch cows were dehorned the after-quantity of milk did not diminish.

*Cross-examined:* Fever sometimes resulted from the operation.

Mr. James McKinny, V.S., Dublin, deposed that he considered the practice of dehorning existed largely in Ireland, and to be increasing as people found the advantage of it. He considered that the horns cut off the defendant's cattle had been properly removed. The pain of dehorning was momentary, and the operation could be performed in a few seconds. There was not much after-pain when the operation was properly performed. In his opinion the animals were improved by having their horns removed. He was not acquainted with any substitute for dehorning; tipping was only a temporary remedy. The cutting away of budding horns from calves was rather dangerous, as the operation was a delicate one.

In cross-examination, the witness said that dehorning caused a little more pain than disbudding. It would be better to disbud each calf if the practice was necessary. In all cases the wounds left after cutting off the horns should be dressed. He believed dehorned cattle to be of more value than polled cattle.

Mr. Thomas Greaves, ex-President of the Royal College of Veterinary Surgeons, Manchester, and Fellow of the same College, and Consulting Veterinary Surgeon to the Society for the Prevention of Cruelty to Animals, said he had believed at one time it was sufficient to dehorn fighting cattle, but he had found that when the bully was dehorned another bully took his place and kept up the unrest in the herd. He believed dehorning to be reasonable and humane when done properly. The pain was trifling and only lasted for a few days. Dehorned cattle could not feed beside horned animals. He was not acquainted with any practical substitute for cutting off the horns.

*Cross-examined:* The result would be the same to take off the budding horns from a calf as dehorning. He had opposed dehorning until a meeting held in Edinburgh six months ago, when he was convinced that the dehorning of cattle was necessary.

Mr. Freeman, V.S., Dublin, deposed that he did not consider the operation cruel, but regarded it as reasonable and justifiable. After the operation there was no diminution of milk and no elevation of temperature. In cross-examination, he stated that the horn in a young calf could not be moved with the finger.

Mr. Reilly, V.S., Navan, also gave evidence, and in cross-examination deposed that for a week after the operation of dehorning the animal was in a bad condition.

Professor Williams, Professor of Pathology in the New Veterinary College, Edinburgh, also gave evidence. In cross-examination, he deposed that in the case of a calf there was bone attaching the base of the horn to the skull. The horn could not be moved about by the finger.

Mr. Simcox, V.S., Drogheda, who saw the defendant's cattle a month after the operation, deposed that he was of opinion that the operation in each case was performed skilfully.

The case for the Crown being closed, Mr. Hart asked permission to go into rebutting evidence in reference to the disbudding of calves. It had been sworn for the defense that the bud was fixed to the skull, and he would reproduce Professor Walley to give evidence on that point. He contended that when the animal was a calf the operation could be performed with the infliction of comparatively no pain.

Mr. Ross objected to a rebutting evidence.

Mr. Henn, R.M., said that Professor Walley had already sworn that the budding horn could be moved with the fingers and could be easily "disbudded." He would, however, take a note of the application.

On Saturday, as had been expected, the summons was dismissed, the magistrates, however, consenting to state a case for the Queen's Bench Division of the High Court of Justice in Ireland.

In delivering the decision of the bench, Mt. Henn (having remarked that the various authorities which had been quoted by counsel on both sides were as conflicting as tne evidence which had been brought before them), said: "We are of opinion from the evidence that the operation of dehorning causes considerable pain, and may in individual cases cause great after-suffering to the animal. We are further of opinion that the defendant had no intent to cause pain in performing the operation, but that he so acted honestly, believing it to be for the benefit of the animals and of himself as a grazier. We are satisfied on the evidence that the dehorning of cattle increases their market value, renders them more salable, is customary and if suppressed would inflict very large pecuniary loss on the community. Cattle, when dehorned, appear to thrive better, to become quiet and easily handled, less dangerous to men and each other and less liable to suffering on pasture and when traveling. That the practice of dehorning has been carried on to a considerab e extent in parts of Scotland, and at least in one county in England, and that it is widely prevalent and daily increasing in Ireland, has been made plain to us. We believe that the straw-yard feeding, both in open and closed yards, is reasonable and necessary, and that as practiced in Ireland is advantageous to the animal, and would be seriously injured by the suppression of dehorning; that there does not appear to be any other practicable method causing less pain by which the same objects sought to be attained can be carried out, provided that it be skilfully performed. We consider that the advantages attainable by the operation of dehorning are out of proportion to the pain suffered by the animal. We find as a matter of fact that in this case the operation was performed with reasonable skill and care, causing at the same time a certain degree of pain, for an adequate and reasonable object not otherwise attainable. Having thus found the facts of the case as disclosed by the evidence, it would follow that we are of opinion that the case should be dismissed on the merits. We consider that the defense of justification should prevail, notwithstanding the recent decision in the case of Forde *vs.* Wiley; and, having regard to the serious conflict of authority which exists on this subject and its paramount importance to large sections of the community, we have, in the best exercise of our judgment, decided to dismiss the case on the merits.

